# Wealth and Family Formation: Insights from First Cohabitation, Marriage, and Birth in Germany

**DOI:** 10.1007/s10680-025-09738-6

**Published:** 2025-07-01

**Authors:** Philipp M. Lersch

**Affiliations:** 1 DIW Berlin, Mohrenstr. 58, 10117 Berlin, Germany; 2 Einstein Center Population Diversity, Berlin, Germany; 3 Humboldt-Universität zu Berlin, Berlin, Germany

**Keywords:** Family, Wealth, Stratification, Event history analysis, Fertility

## Abstract

Existing research has demonstrated that wealthier individuals differ in family formation. Potential explanations draw on wealth’s use and symbolic value as well as the relative economic bar of family formation. This study examines the relationship between wealth and three family formation events in Germany: first cohabitation, marriage, and birth. Data from the German Socio-Economic Panel Study (2002–2017) are used with multi-state, competing-risk, discrete-time event history analysis. Results show that wealth, primarily driven by homeownership, significantly influences cohabitation, marriage, and birth. The impact of homeownership is particularly notable for marriage and birth and shows gender-specific associations for cohabitants. The findings highlight the substantial influence of wealth—most likely through its symbolic and use value—in shaping family formation while indicating limited support for a relative economic bar in Germany.

## Introduction

There is a long tradition of demographic research on the economic foundations of family formation, i.e. the transition to first cohabitation, marriage, and childbirth (Kreyenfeld, 2010). Much of this research is concerned with the role of economic conditions related to the labour market, such as income and employment insecurity (e.g. Meggiolaro et al., 2024; Van Wijk & Billari, 2024), for family formation. Moving beyond narrow conceptions of economic conditions, recent research identifies individuals’ economic wealth as an essential and distinct resource in family formation (Su & Addo, 2023). Building on earlier ethnographic findings (Edin & Kefalas, 2011), a growing body of survey-based evidence shows that the wealthy tend to differ in whether and when they cohabit, marry, and have children to form families (e.g. Addo, 2014; Schneider, 2011; Tocchioni et al., 2021).

Understanding how wealth affects family formation is relevant for at least two reasons. First, wealth inequality can hinder some from forming a family because they (subjectively) lack the necessary resources. In turn, selective family formation may intensify wealth concentration in the next generation, where wealth is an essential and distinct dimension of social stratification (Killewald et al., 2017). Second, it is argued that economic shifts and growing economic inequality may have contributed to fundamental changes in family formation in affluent countries in recent decades, for example, by delaying childbearing and significantly worsening the chances of low-educated men in the partner market (McLanahan, 2004). However, in explaining these changes, previous research mainly focuses on shifts in the labour market and income inequality (Sassler & Lichter, 2020), largely ignoring a parallel trend of rising wealth inequality following World War II in many countries (Piketty & Saez, 2014).

There are three main complementary explanations for why wealth may matter for family formation. First, wealth provides use value, i.e. wealth can satisfy individuals’ needs and wants. Wealth can buy things and wealthy people are more likely to be able to afford to move in together, get married, and have children without economic strain. Second, it is argued that wealth has symbolic value. Specific beliefs and ideas exist about which assets must be owned to form a family (Schneider, 2011). Third, and closely related, meeting a relative standard of wealth—the economic bar of family formation—may be perceived as necessary because of cultural norms of middle-class financial achievement and readiness for forming a family (Ishizuka, 2018). While these explanations underscore the multi-faceted role of wealth in family dynamics, it proves challenging to empirically disentangle them due to the intertwined nature of wealth’s use value, symbolic value, and relative wealth standards, and they have not been jointly examined in prior research. Nevertheless, careful interpretation of empirical evidence may suggest the relative importance of these explanations.

This study improves on prior literature by examining the relationship between the multiple facets of wealth and three family formation events in Germany: first cohabitation, marriage, and birth. I address the following research question: Are wealthier people more likely to experience first cohabitation, marriage, and birth than the less wealthy in Germany? I draw on the German Socio-Economic Panel Study (SOEP 2002–2017; *N* = 8,830 individuals) and discrete-time event history models accounting for multiple states and competing risks in the family formation process in answering this question. While I cannot estimate the separate causal effect of wealth’s use value, symbolic value, and relative wealth standards on family formation with the observational data from the SOEP, the data allow for capturing wealth’s multiple facets and accounting for relevant alternative explanations, such as the influence of family of origin, labour market characteristics, and risk preferences.

## Background

### Theory

*The use value of wealth for family formation*. Moving in together, getting married, and having children are all associated with financial costs—including opportunity costs of foregone earnings for those (primarily women) caring for children. Because wealthy people can afford these costs of family formation, they are more likely to move in together, get married, and have children without economic strain, according to the use value argument (Schneider, 2011). Furthermore, wealth provides long-term financial security and stability and safeguards against unexpected economic shocks through the insurance function of wealth. For instance, in the case of unemployment or severe illness, wealth can provide an additional, flexible safety net beyond public welfare provision.

Wealth is often narrowly conceptualized as net wealth, the difference between privately owned assets (gross wealth) and debts. However, recent scholarship calls for distinguishing gross wealth from debt as two distinct aspects of wealth (Dräger et al., 2023; Su & Addo, 2023). Gross wealth is the most relevant measure of wealth’s use value, because it captures the full potential of wealth to satisfy individuals’ needs and wants through divestiture (Schneider, 2011). Even when not sold, gross housing wealth, i.e. the value of the primary residence, also indicates use value because the higher gross value is typically associated with superior accommodation in larger, higher-quality homes and better neighbourhoods. Homeownership is also associated with housing security in many cases (Vignoli et al., 2013) and may reduce housing costs in the long run (Lersch & Dewilde, 2018). Debt’s role in use value is more ambivalent. On the one hand, debt can reduce the economic resources available to individuals because of repayments. On the other hand, debt, particularly when secured, can signal economic potential.

The importance of wealth’s use value may differ between entering first cohabitation, marriage, and parenthood. For example, moving together into an apartment to cohabit can be costly initially. Savings may cover these one-off costs. Those with savings may also be more attractive as partners and, therefore, more likely to cohabit. However, moving in together also creates economies of scale, which may reduce costs and motivate the economically disadvantaged to cohabit. Marriage is associated with moderately higher costs, such as buying engagement and marriage rings alongside paying for a wedding. Having children is associated with the highest economic costs, ranging from buying food and clothes to paying for childcare and education. In Germany, families with one child spent about EUR 760 a month on their child in 2018 (Statistisches Bundesamt, 2021). Economic security offered by wealth may be seen as a prerequisite for marriage and, even more so, having children (Kreyenfeld, 2010; Schneider, 2011; Su & Addo, 2023). Because expectations about economic security are less entrenched in cohabitation (Hiekel et al., 2014), wealth may play a less critical role in transitions into first cohabitation.

*The symbolic value of wealth for family formation*. A complementary explanation for the relevance of wealth in family formation builds on the symbolic value of wealth. Wealth can signal readiness linked to schemas of necessary achievement and social status for family formation. Visible assets like a home may convey that one has arrived at a point where essential aspects of an individual’s life are settled (Killewald et al., 2023). Owning these assets aligns with popular schemas of the ideal setting for marriage and serves as an “economic litmus test” for couples (Gibson-Davis et al., 2018). In contrast, less visible assets, such as savings, provide little symbolic value. Passing this litmus test may also generally increase the probability of having children, but less so than for marriage. Marriage and fertility have become increasingly disconnected in recent decades, and while marriage has gained symbolic importance because it is less frequent and increasingly exclusive, the same symbolic importance is not assigned to fertility (Gibson‐Davis, 2011).

The symbolic value of wealth, particularly homeownership, can be expected to be of limited importance for cohabitation. Cohabitation may be the first step in forming a family and can be a durable alternative to marriage (Sassler & Lichter, 2020). Cohabitation, however, may not draw on the symbolic value of wealth because cohabitation is not laden with cultural expectations about economic achievement and security (Hiekel et al., 2014). Furthermore, cohabitation is commonplace in modern romantic relationships among young adults, who typically lack wealth (Addo, 2014).

*The relative bar of family formation*. The relative wealth position of individuals, i.e. how much wealth they own compared to others, could be a relevant indicator of whether they meet the norms for family formation linked to “middle-class standards of living” (Ishizuka, 2018, p. 540). The relative wealth position builds on the idea that individuals compare themselves to significant referents in evaluating their financial situation. This idea is incorporated in the literature on relative income and family formation (e.g. Ishizuka, 2018) but not yet in the literature on wealth. For instance, Easterlin (1987) argues that individuals’ current income compared to their parents’ income during childhood and adolescence influences marriage and fertility decisions. Similarly, it may be argued that individuals will postpone marriage and childbirth until they have reached similar levels of wealth relative to their parents because this marks their economic aspirations.

Instead of comparing one’s situation to past experiences, other studies emphasize other referents in the current economic environment. For instance, studies relate an individual’s income to the current median value of a reference group with similar characteristics (Ishizuka, 2018). Because wealth strongly depends on age (Killewald et al., 2017), I consider individuals in the same age group born around the same time to provide a relevant reference for individuals’ economic achievement. Thus, I argue that individuals may compare themselves to others in their birth cohorts regarding whether they arrived at a sufficient financial standing. It follows that if individuals are relatively well-off compared to others in their birth cohorts—for instance, if they are above the median wealth—they should be more likely to marry and have children regardless of their absolute level of wealth because they are “on track.” Again, because cohabitation is less associated with economic standards, the relative wealth position may matter less when entering cohabitation.

*Similar relationships for women and men*. Starting with Oppenheimer (1988), it is argued that economic resources have a similarly positive effect on family formation for women and men. Women’s resources are equally crucial for sustaining an economically secure household. Women’s contribution is particularly important when the labour market income of one (male) household member may no longer sufficiently provide for the whole family. Thus, following this argument, wealthier individuals are more attractive, but they may postpone their decisions to cohabitate, marry, and have children as their resources allow them to extend their search for the right partner (Sweeney, 2002; Xie et al., 2003). While these arguments mainly refer to wealth’s use value, initial empirical evidence also finds wealth’s symbolic value to matter similarly for women and men (Schneider, 2011). Therefore, I expect the relationship between wealth and family formation to be similar for women and men.

*Interdependent events*. Beyond the multi-faceted nature of wealth, exploring the association between wealth and family formation is complicated by their mutual interdependence. For instance, entry into homeownership may occur in anticipation of future births, or in other words, the decision to buy and the decision to have children are choices that individuals may make concurrently (Öst, 2011; Tocchioni et al., 2021). In addition, family formation events are closely linked such that person-specific influences on the transition to cohabitation are likely correlated with transitions into marriage and fertility (Mikolai & Kulu, 2022).

*Alternative explanations*. A spurious relationship between wealth and family formation may arise regardless of wealth’s use, symbolic value, and the relative bar of family formation. First, the socio-economic status in the family of origin significantly influences family formation behaviour (Billari et al., 2019). At the same time, the family of origin is crucial for individuals’ wealth through direct transfers and inheritances as well as indirect relationships such as the transmission of investment behaviour (Lersch & Groh-Samberg, 2023). Second, labour income and labour market position are essential determinants of wealth accumulation (Killewald et al., 2017) and are also linked to family formation (Kreyenfeld, 2010). Third, personality traits, such as being risk-averse, are simultaneously associated with wealth and family formation processes (Brown & Taylor, 2014; Schneider, 2011).

### Prior Empirical Evidence on Wealth and Family Formation and its Shortcomings

While labour market conditions, such as income, have received considerable attention in explaining family formation (e.g. Meggiolaro et al., 2024; Van Wijk & Billari, 2024; Van Wijk et al., 2021), wealth is generally less studied. Preliminary evidence supports the idea that the use value and symbolic value of wealth are related to family formation, with most evidence regarding marriage in the USA. For instance, ethnographic studies indicate that poorer individuals consider wealth a necessary prerequisite for marriage because of its symbolic value captured by ownership status in the USA (Edin & Kefalas, 2011; Gibson-Davis et al., 2005). According to survey findings, absolute net wealth and possessing certain assets are associated with the likelihood of entering into a first marriage in the USA. For men, this includes owning a car and financial assets, while for women, it involves having a car and other assets (excluding home ownership). This correlation is seen as indicative of wealth’s symbolic and use value, with the understanding that a simple ownership measure tends to emphasize symbolic value more than use value when compared to overall wealth (Schneider, 2011). There is no association between, on the one hand, net wealth and wealth ownership of a home or car and, on the other hand, first childbirth in the USA (Su & Addo, 2023).^1^ In studies that do not consider other aspects of wealth, homeownership status is positively associated with marriage in the USA (Gibson-Davis et al., 2018; Ishizuka, 2018) and Sweden (Holland, 2012), but there are also findings of null (Netherlands) and negative associations (Germany) (Mulder et al., 2006). In Germany, entry into homeownership often occurs after marriage (Mulder & Wagner, 2001).

The relationship between homeownership and fertility (without accounting for other aspects of wealth) is more intensively studied (Chudnovskaya, 2019; Japaridze & Sayour, 2024; Kulu & Steele, 2013). For instance, there is evidence of a positive association between homeownership status and first births in Germany (Mulder & Wagner, 2001). A similar positive association in Britain has decreased in size in recent years (Tocchioni et al., 2021). Importantly, residential relocations and entry into homeownership also occur in anticipation of future births (Kulu & Steele, 2013; Vidal et al., 2017). Relatedly, fertility may be delayed until homeownership is achieved. However, fertility can also delay entry into homeownership due to the competing costs of these transitions (Tocchioni et al., 2021).

While the literature generally supports a relationship between wealth and family formation, I identify three significant shortcomings. First, there are few attempts to illuminate the different explanations for why wealth may matter for family formation events in recent years. Second, previous studies did not test the relative bar of family formation regarding wealth in contrast to the literature on income and family formation (e.g. Ishizuka, 2018). Third, no previous study includes cohabitation and few studies on wealth beyond homeownership consider childbirth. Finally, most existing literature draws on data from the USA, a society that stands out through its stark economic divisions in family behaviour (Cherlin, 2020; Sassler & Lichter, 2020). It is unclear whether these results would hold in less divided societies such as contemporary Germany.

### German Context

Wealth is highly unequally distributed in Germany in international comparison, but wealth inequality is significantly lower compared to the USA (Pfeffer & Waitkus, 2021). Germany remains a developed welfare state despite fundamental restructuring and retrenchment in recent decades (Seeleib-Kaiser, 2016). The welfare state is reflected in the much lower post-tax and post-transfer income inequality in Germany compared to the USA (OECD, 2023a). The German welfare state also supports families through, amongst other transfers, universal child benefits, means-tested parental leave benefits, and subsidized childcare to reduce the economic costs of children for parents (Gangl & Ziefle, 2015). Joint ownership of assets is common within couples, in particular when married, but gender inequality in wealth is still substantial with women owning less wealth, on average (Grabka et al., 2015; Nutz, 2022).

Germany has one of Europe’s lowest homeownership rates, with 42 per cent in 2022 (Statistisches Bundesamt, 2023a). Homeownership is a significant life investment for most German households and down payments are relatively high due to strict mortgage regulations. As a result, families need to save for a long time before buying a home (Thomas & Mulder, 2016). However, since the 2000s, there has been substantial house price inflation, with real house price indices increasing by 40 per cent between 2015 and 2022 (OECD, 2023b), particularly in urban areas. Germany has an attractive and—in international comparison—inexpensive and strongly regulated rental sector (Voigtländer, 2009). Mulder and Billari (2010) categorize Germany as a “Career Homeownership Regime” where homeownership is a potential, but neither universal nor normative, step in the housing career for those with a stable economic perspective.

Overall, the economic division of family structure in Germany is less pronounced compared to the USA, but divisions are more prominent than in other European countries. For instance, entry into marriage is more strongly associated with men’s education (not women’s) in Germany compared to most other European countries (Kalmijn, 2013). Strong negative gradients of education and wages in women’s fertility exist in Germany compared to France (Lipowski et al., 2022). A higher educational level increases the likelihood of marriage when the first child is born in Germany (Perelli-Harris et al., 2018).

In Germany, cohabitation rates have increased, and marriage rates have declined in recent decades, but social policies and tax laws still favour marriage over cohabitation (Perelli-Harris et al., 2018). For example, tax splitting and sharing the health insurance of the primary earner are only available to married couples. Despite shared institutional and political conditions since reunification in 1990 and the alignment of other family behaviours, such as fertility and divorce, the eastern and western parts of the country still differ considerably when it comes to the prevalence and meaning of cohabitation. For example, birth rates among cohabiters are markedly higher in Eastern compared to Western Germany (Perelli-Harris et al., 2018). Fertility rates in Germany are very low, with a total fertility rate of 1.46 in 2022, but similar to the European average (Statistisches Bundesamt, 2023b).

### Expectations

Table 1 summarizes the expectations. Based on theory and previous empirical evidence, I generally expect to find a positive relationship between wealth and family formation. For each of the theoretical explanations outlined above, I suggest one key variable to test the explanation. However, it is important to note that these variables are not exclusively related to one explanation. For example, homeownership is the primary variable to test the symbolic value of wealth, but homeownership is also associated with substantial use value.

**Table 1:** Summary of expectations

Theory	Key explanatory variable	Expected effect on chances to experience		
		Cohabitation	Marriage	Birth
Use value	Gross wealth	+	+ +	+ + +
Use value	Debt	?	?	?
Symbolic value	Homeownership	0	+ +	+
Relative bar	Above-median net wealth	0	+	+

The number of pluses indicates the expected strength of the relationship relative to the other family formation events for the same explanatory variable. Pluses do not indicate expectations regarding the strength of the relationship across explanatory variables. The explanatory variables are described in detail in the Measurement section

More specifically, I expect gross wealth to be positively associated with entry into cohabitation, marriage, and parenthood in Germany, mainly because of gross wealth’s use value. The positive association should be strongest for childbirth, followed by marriage, and then cohabitation, as the financial costs of these life events decrease in that order. I expect homeownership, as a symbolic marker of economic achievement, to be positively associated with entry into parenthood and, even more so, marriage. I expect above-median net wealth to be positively associated with entry into marriage and parenthood because of the relative economic bar and schemas of middle-class living standards relevant to these family formation events but not cohabitation. Because of its ambivalent nature, I cannot formulate a clear expectation for debt. Finally, these expectations should hold for women and men. However, because the age patterns and dynamics of family formation differ strongly for women and men, I follow standard practice in the literature (e.g. Schneider, 2011) and test expectations separately for women and men.

## Data and Methods

**Fig. 1:**
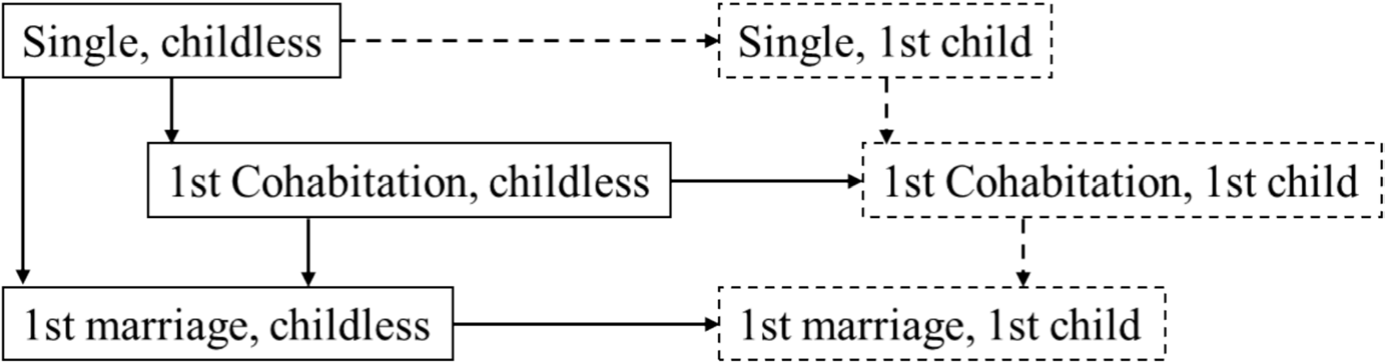
Family formation states and transitions. *Note:* Own elaboration adapted from Mikolai and Kulu (2022, Fig. 1). Boxes indicate family states. Arrows indicate transitions. Dashed boxes are included as outcomes of transitions in the analysis, but not as origin family states due to few respondents observed in these states (single and cohabitation with one child) and focus on early family formation (marriage). Dashed lines indicate transitions that are not examined due to small case numbers in the data

I used longitudinal panel data, including retrospective family biographies, with competing-risk, discrete-time event history analysis to test the expectations for events of cohabitation, marriage, and childbirth accounting for diverse origin states (single, cohabiting, married). Discrete-time event history models are widely used in research on union formation and fertility (e.g. Tocchioni et al., 2021). Figure 1 shows the family states and transitions I studied, following Mikolai and Kulu (2022). I focussed on the first transitions in the family formation process and did not consider union dissolution, repartnering, or higher-order births.^2^ In addition, because of the small case numbers in the SOEP, I did not consider transitions from childless single to single with a first child and subsequent transitions for singles with a first child and those in their first cohabitation with a first child.

### Data

I used longitudinal data from the German SOEP (version 38; https://doi.org/10.5684/soep.core.v38eu), an extensive, nationally representative household panel survey (Goebel et al., 2019). All adult household members were individually interviewed, and one household member provided additional information on the household. Individual-level wealth was recorded in 2002, 2007, 2012, and 2017, allowing for capturing young people’s wealth separately from their parents’ wealth if in the parental home. I used all 16 waves between 2002 and 2017 as well as all subsamples for which at least two wealth measurements were available.^3^ I constructed a longitudinal dataset in which each observation represented a single individual-year nested within individuals.

### Samples

Following Mikolai and Kulu (2022), I worked with three different analytical samples based on the current family state of respondents (see solid boxes in Fig. 1) because individuals were exposed to fundamentally different transition risks across family states. The first sample included never-married, childless singles who had not experienced cohabitation in the past (childless single state).^4^ In this sample, individuals were at risk of experiencing first cohabitation and first marriage (first childbirth for singles was not considered due to small case numbers). The second sample included never-married, childless individuals in their first cohabitation who were at risk of experiencing first marriage and birth (childless cohabiting state). The third sample included childless individuals in their first marriage who were at risk of experiencing first childbirth (childless married state). Respondents could be included in more than one sample. I separated the samples by gender for all analyses, given the differential timing of demographic transitions for women and men.

All samples were restricted to individuals in private households born after 1959 and aged 18–44 from 2002 to 2017.^5^ I imposed the age restriction in line with previous research to focus on young adults most likely to experience family formation (Schneider, 2011; Tocchioni et al., 2021). I right-censored all samples at event occurrence, age 44, in 2017, or at permanent panel drop-out in earlier years, depending on what happens first. All samples were left-truncated for those respondents who entered the SOEP after age 18, but I could draw on retrospective family histories for these respondents. The wealth measures used in the current study (see below) were multiply imputed with five sets of values by the SOEP survey team (Grabka & Westermeier, 2015).^6^ For other analytical variables, I used listwise deletion.

I observed 2,984 women who experienced 482 cohabitation events and 97 marriage events alongside 3,400 men who experienced 426 cohabitation events and 75 marriage events in the sample for the childless single state. I observed 964 women who experienced 179 marriage events and 150 birth events alongside 816 men who experienced 144 marriage events and 110 birth events in the sample for the childless cohabitation state. Finally, in the childless marriage state sample, I observed 988 women who experienced 423 birth events alongside 871 men who experienced 364 birth events.

### Measurement

*Outcome variables*. The outcomes of interest were the transitions into first cohabitation, first marriage, and first birth (I only considered live birth due to data limitations). For example, the first outcome indicated whether a first cohabitation entry occurred (coded 1) or not (0) within the period from the current interview until the following interview. The first marriage and first birth event outcomes were constructed similarly. I built these variables using retrospective life history and prospective panel data from the SOEP. Thereby, I could determine whether the observed event is the first to occur in individuals’ biographies. To identify first cohabitations, I used retrospective cohabitation histories, which were only available for respondents who had answered a biography questionnaire since 2011 or entered the SOEP at age 17. For first marriages, I used retrospective marriage histories available for all respondents. For first births, I used retrospective birth histories, which are only available for men if they entered the SOEP after 2000.

*Explanatory variables*. The explanatory variables measuring wealth were observed in the wave before an event potentially occurred. I differentiated between gross wealth—ranging from real assets, financial assets, life insurance, private pension plans, business assets, to valuable assets such as jewellery—and debt—including mortgages and consumer debt. Both variables were price-adjusted (in 2015 EUR). I then used an inverse hyperbolic sine transformation, which is similar to a log transformation but is defined for 0 values, to account for the skewed distribution and to pull in outliers (Friedline et al., 2015). I added indicators of homeownership (1 = yes, 0 = no) and whether respondents had above-median net wealth (gross wealth minus debts) in reference to their birth cohort in a given survey year (1 = yes, 0 = no).^7^ I grouped respondents in eight quinquennial birth cohorts: 1960–1964, 1965–1969, 1970–1974, 1975–1979, 1980–1984, 1985–1989, 1990–1994, and 1995–1999. All wealth variables were measured at the individual level and included assets solely owned by an individual and the individually owned share of assets jointly owned with other household members. I preferred individual-level measures for the primary analysis because they allowed for capturing young people’s wealth even if they lived in the parental home. I also report additional results using household-level wealth in Online Appendix C.

To construct the explanatory variables, in the first step, I linearly interpolated wealth at the individual level between 2002, 2007, 2012, and 2017 to fill in the years between the two measurements. I did this only for individuals observed in both adjacent years. For individuals with only one observation, wealth was not interpolated. For instance, I filled in wealth for 2003, 2004, 2005, and 2006 for individuals observed in 2002 and 2007, but I did not fill in wealth for individuals only observed in 2002 and not in 2007.^8^

The four explanatory variables were introduced to capture the different aspects of wealth presented above. However, empirically, there will be an overlap between these variables that must be considered in interpretation, a challenge also faced in other studies on the relationship (e.g. Schneider, 2011). For instance, those in homeownership will have more gross wealth, more debt (due to mortgages), and will be more likely to have above-median net wealth, on average. Indeed, the bivariate correlations between the explanatory variables are substantial (Table B.2 in Online Appendix B). Because of this overlap, isolating the effect of each variable is difficult. Still, variance inflation factors as a measure of multicollinearity for the four wealth variables from linear regression models are all below 4 and mostly below 3, indicating only moderate multicollinearity between the wealth variables in the model (James et al., 2021, p. 102). I first estimated models with each explanatory variable included separately. Then, I included the variables jointly in one model.

*Covariates*. I broadly based the selection of covariates on previous literature (Addo, 2014; Schneider, 2011) and considered potential confounders introduced above. I included the following time-constant covariates: *number of siblings, mother’s age at birth, parents’ highest education* when respondents were aged 15 (low [no formal education or level 1 and 2 in the International Standard Classification of Education (ISCED)], intermediate [ISCED-level 3 and 4; reference], and high [ISCED-level 5 and 6]), *migrant status* (1 = yes [including second generation migrants], 0 = no;), *birth cohort* (collapsed into four groups due to small sample size: 1960–1969, 1970–1979, 1980–1989, and 1990–1999), and *subsample membership* (see Footnote 3). I included time-constant, individual-specific averages of the following covariates: *risk preferences* and *frequency of religious services*. I included the following time-variant covariates, which were recorded before an event may occur: *self-rated health, education* (low [no formal education or level 1 and 2 in the International Standard Classification of Education (ISCED)], intermediate [ISCED-level 3 and 4; reference], and high [ISCED-level 5 and 6]), price-adjusted, annual, individual labour income (log-transformed), *temporary employment* (1 = yes, 0 = no), *currently unemployed* (1 = yes, 0 = no), *part-time employed* (1 = yes, 0 = no), *self-employed* (1 = yes, 0 = no), *lives in the parental home* (1 = yes, 0 = no), *urban area* (1 = yes, 0 = no), *Eastern Germany* (1 = yes, 0 = no). In addition, I adjusted for the duration of the partnership in years in the samples for the cohabitation and marriage state. See Table B.1 in Online Appendix B for an overview of all analytical variables.

### Method

I used a multi-state, competing-risk, discrete-time event history model (Mikolai & Kulu, 2022) because family formation events in the SOEP were only recorded in yearly time intervals. In addition, respondents could experience competing events of family formation originating from different family states. Therefore, I calculated hazard rates of competing events for each yearly time interval using a multinomial logistic regression framework separately for origin states (Steele, 2011). Respondents entered the risk set at age 18. I right-censored at age 44. Years since age 18 were used as the primary clock for the event history analysis (but I also adjusted for partnership duration; see above).^9^

Generally, the model is written as follows:

1 $${\varvec{log}}\left( {\frac{{{\varvec{h}}_{{\varvec{i}}}^{{\left( {{\varvec{r}},{\varvec{s}}} \right)}} \left( {\varvec{t}} \right)}}{{{\varvec{h}}_{{\varvec{i}}}^{{\left( {0,{\varvec{s}}} \right)}} \left( {\varvec{t}} \right)}}} \right) = {\varvec{\alpha}}^{{\left( {{\varvec{r}},{\varvec{s}}} \right)}} \left( {\varvec{t}} \right) + {\varvec{\beta}}^{{\left( {{\varvec{r}},{\varvec{s}}} \right)}} {\varvec{x}}_{{\varvec{i}}}^{{\left( {{\varvec{r}},{\varvec{s}}} \right)}} \left( {\varvec{t}} \right) + {\varvec{\delta}}^{{\left( {{\varvec{r}},{\varvec{s}}} \right)}} {\varvec{y}}_{{\varvec{i}}}^{{\left( {{\varvec{r}},{\varvec{s}}} \right)}}$$

where subscript *i* identifies individuals and *t* identifies yearly time, *h*_*i*_ is the hazard rate of forming a family estimated for competing events *r* (first cohabitation, marriage, and birth) in separate models for states *s* (childless single, childless cohabiting, childless married). To reduce model complexity, I follow previous literature in examining origin states separately (Billari et al., 2019; Mikolai & Kulu, 2022). The sets of *r* vary over *s*. *α(t)* is the piecewise constant baseline hazard fitted using a step function for ages 18–24, 25–29, 30–34, and 35–44. ***x***_***i***_ is a vector of time-varying variables, particularly the main explanatory variables that capture gross wealth, debt, homeownership, and above-median net wealth, with the related coefficients ***β***. Note that all time-varying variables are measured at the time of the interview (labour income is measured for the calendar year before the interview) and preceded any family formation event measured in the yearly time interval between the current until the next interview. ***y***_***i***_ is a vector of time-constant variables, and ***δ*** are the associated coefficients. I made the proportional hazard assumption, i.e. the effects of covariates were assumed to be constant over *t*. Because I drew on observational data, the estimates of ***β*** and ***δ*** are biased if additional, unobserved factors are related to ***x*** or ***y*** and associated with family formation beyond the broad range of included covariates. In further analyses reported in Online Appendix D, I follow Öst (2011) in estimating event history models of family formation simultaneously with models of homeownership entry to address concurrent family formation and housing decisions. My general conclusions are consistent. For the primary analysis, I report results as average marginal effects (AMEs), i.e. the percentage-point change in the predicted probability of an event occurring vs. no event occurring for a given characteristic. I used Stata 17 to estimate all models (StataCorp, 2021).

## Results

**Fig. 2:**
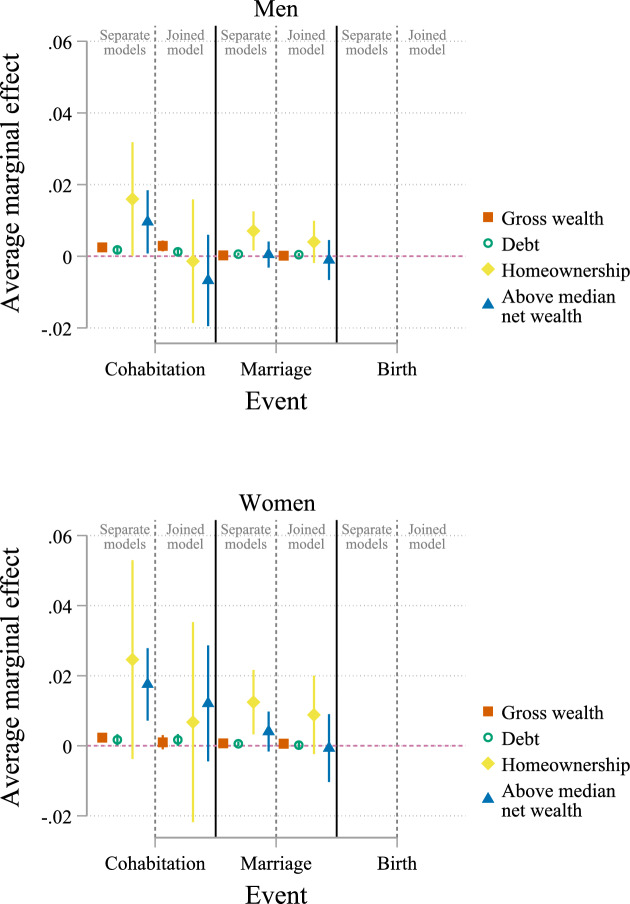
Average marginal effects of wealth for state childless single. Data: SOEP v38 (unweighted, imputed). *Notes*: Based on multinomial logistic regression with covariates: age, number of siblings, mother’s age at birth, parental education, immigrant, self-rated health, education, labour income, temporary employment, currently unemployed, part-time employed, risk preferences, monthly religious service, lives in parental home, Eastern Germany, urban, and subsample membership; cluster robust standard errors. Full estimation results are available in Tables B.3, Online Appendix B

**Fig. 3:**
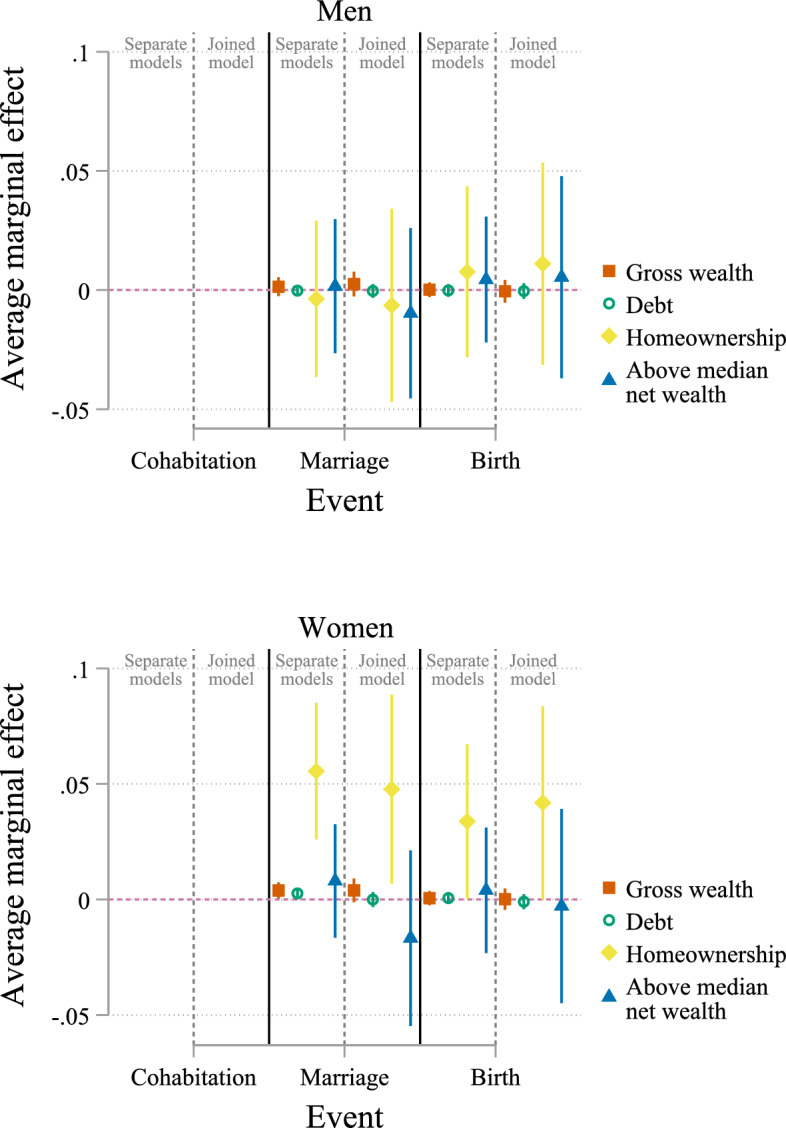
Average marginal effects of wealth for state childless cohabiting. Data: SOEP v38 (unweighted, imputed). *Notes*: Based on multinomial logistic regression with covariates: age, number of siblings, mother’s age at birth, parental education, immigrant, self-rated health, education, labour income, temporary employment, currently unemployed, part-time employed, risk preferences, monthly religious service, lives in parental home, Eastern Germany, urban, partnership duration, and subsample membership; cluster robust standard errors. Full estimation results are available in Tables B.4, Online Appendix B

**Fig. 4:**
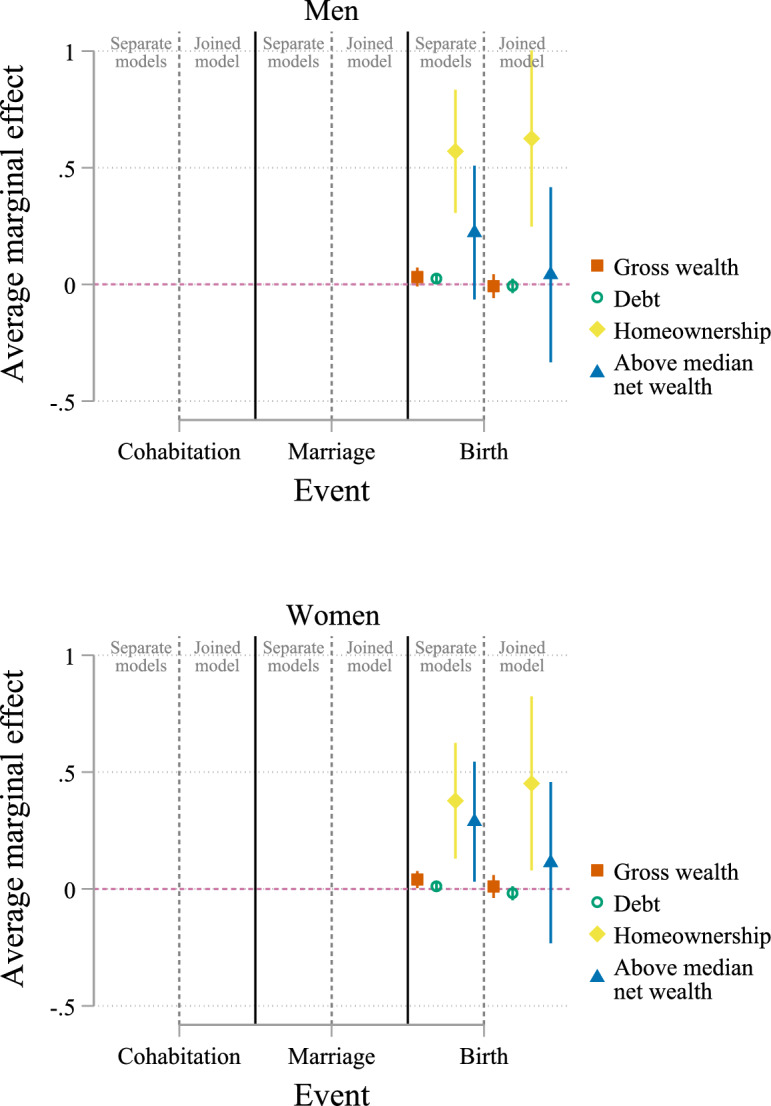
Average marginal effects of wealth for state childless married. Data: SOEP v38 (unweighted, imputed). *Notes*: Based on multinomial logistic regression with covariates: age, number of siblings, mother’s age at birth, parental education, immigrant, self-rated health, education, labour income, temporary employment, currently unemployed, part-time employed, risk preferences, monthly religious service, lives in parental home, Eastern Germany, urban, partnership duration, and subsample membership; cluster robust standard errors. Full estimation results are available in Tables B.5, Online Appendix B

Online Appendix A presents bivariate, descriptive results. Figures 2, 3 and 4 show AMEs for wealth from discrete-time event history models in a (multinomial) logistic regression framework. The explanatory variables gross wealth, debt, homeownership, and above-median net wealth are tested separately and then in a joined model. The Online Appendix B shows full estimation results in Tables B.3–B.5. The interpretation of the direction of effects and statistical significance based on logit coefficients in these tables is consistent with my interpretation of AMEs.

In separate models, I find evidence for a positive association between gross wealth, debt, homeownership, and above-median net wealth with the transition to cohabitation for single women and men (Fig. 2). Although the estimated effect is large for homeownership among women, the effect is not statistically significant. For instance, a 1-per cent increase in gross wealth increases the probability of cohabitation by 0.3 percentage points for men and 0.2 percentage points for women. Having above-median net wealth increases the likelihood of cohabitation by about 1.0 percentage points for men and 1.8 percentage points for women. Moving from separate models to a joined model in which all explanatory variables are included reduces estimated effect sizes considerably, and most AMEs turn statistically insignificant. For men, only gross wealth and debt remain statistically significant, with a 1-per cent increase in gross wealth increasing the probability of cohabitation by 0.3 percentage points and a 1-per cent increase in debt increasing the probability of cohabitation by 0.1 percentage points. For women, all estimated effects are statistically insignificant in the joined model.

I find positive associations between homeownership and the transition to marriage from childless single in models considering the explanatory variables separately. For childless single men, men who own their homes have a 0.7-percentage-point higher probability of transitioning to marriage (Fig. 2). For women, the probability is 1.2 percentage points higher. Debt is also positively associated with transitioning into marriage for men and women. In joined models, the effect sizes are slightly reduced, and the AMEs are no longer statistically significant. The only exception is a positive association between debt and the transition into marriage for men in the joined model, but the effect size is almost 0. Other wealth variables have no substantial or statistically significant estimated effect on the transition into marriage for single women and men.

For childless cohabiting women, the chances to marry are considerably higher when they are homeowners compared to those renting (Fig. 3). When estimated in a separate model, homeownership is associated with a 5.6-percentage-point higher probability of transition into marriage for women. Considering homeownership in a joined model with the other explanatory variables slightly reduces the estimated strength of the association, but the AME remains statistically significant. Gross wealth and debt are positively associated with the transition into marriage for cohabiting women in separate models, but the estimated effects become insignificant in a joined model. For childless cohabiting men, none of the explanatory variables are associated with the transition to marriage.

Next, I consider first births, i.e. the transition to parenthood. Figure 3 shows that homeownership increases the probability of birth for childless cohabiting women. Being a homeowner is associated with a 3.4-percentage-point higher probability of transitioning to parenthood for women when estimated in a separate model (4.2 percentage points in a joined model). There is no evidence that the other aspects of wealth are associated with birth. For men, gross wealth, debt, homeownership, and above-median net wealth show no statistically significant association with births among childless cohabiters.

For married women and men, homeownership is strongly positively associated with the transition to parenthood (Fig. 4). The effect sizes for homeownership are considerably larger compared to the state childless cohabiting. For women, homeownership is associated with an increased probability of birth by 37.7 percentage points in a separate model (45.1 percentage points in a joined model). For men, homeownership is associated with a 57.0-percentage-point higher probability of birth in a separate model (62.5 percentage points in a joined model). Gross wealth and above-median net wealth are positively associated with births among women in separate models. Still, the estimated effect sizes are considerably reduced in joined models and are no longer statistically significant. For men, debt is positively associated with the transition to parenthood in a separate model, but the estimated effect is statistically insignificant in the joined model.

To summarize, against my expectations, not only is gross wealth not consistently positively associated with entry into cohabitation, marriage, and parenthood, but its association does not increase across family formation events. Homeownership, as a symbolic marker of economic achievement, is mostly positively associated with entry into marriage and parenthood, which is aligned with my expectations. Against my expectations, above-median net wealth is not positively associated with entry into marriage and parenting, with above-median wealth for married women in separate models being the exception. Results are similar for women and men, but I discuss the noteworthy exception for those cohabiting in the conclusion.

Tables B.6–B.8 in the Online Appendix B also show reduced models excluding control variables other than birth cohort and subsample membership. An estimated large and positive effect of above-median net wealth on marriage among childless cohabiting women is reduced by three quarters when accounting for the control variables and turns insignificant. Similarly, estimated positive effects of gross wealth and above-median net wealth on birth among childless married men are reduced by half when accounting for the control variables and turn insignificant. For other origin states and transitions, changes in effect sizes are modest. Control variables generally behave as expected.

In additional analysis reported in Online Appendix C, I considered household-level wealth instead of individual-level wealth.^10^ Generally, differences between household-level measures and individual-level measures of wealth may emerge, for instance, if other household members (parents or partners) own wealth rather than the focal individual. Household-level wealth does not correlate with the transition to cohabitation for single men, contrasting the patterns observed with individual-level wealth measures. Additionally, for single women, household-level homeownership is negatively linked to the transition into cohabitation. These findings are consistent with prior research indicating that parental homeownership reduces the likelihood of leaving the parental nest (Mulder et al., 2002). Similarly, household-level homeownership and above-median net wealth are negatively associated with the transition to marriage for cohabiting men. However, the results align more closely between household-level and individual-level wealth for married men and women.

## Conclusion

Prior literature—mainly from the USA—shows that the wealthy differ in whether and when they cohabit, marry, and have children to form families. For example, net wealth and possessing specific assets are positively associated with the likelihood of entering a first marriage in the USA (Schneider, 2011). These patterns may emerge through the use and symbolic value of wealth and relative wealth standards, but these aspects of wealth have not been jointly examined in prior literature. The current study improves on prior research by considering these explanations across the three family formation events of first cohabitation, marriage, and birth in Germany using high-quality data from the SOEP with discrete-time event history analysis.

The primary insight from this study is the significant role of homeownership in influencing individuals’ transitions to cohabitation, marriage, and parenthood. Although the association between homeownership and cohabitation is somewhat less pronounced compared to other family formation events, it remains statistically significant in a separate model for men. Notably, when examining other wealth factors, such as gross wealth, in separate models, the effect often appears to be largely encapsulated within homeownership when shifting to joined models. This reaffirms the well-established understanding of homeownership’s central role in family formation in demographic research (e.g. Mulder & Billari, 2010). The predictive power of simple homeownership indicators compared to the other wealth measures is good news for researchers because this information is more easily collected than assets’ value.

It is essential to be cautious in interpreting the estimated positive association between homeownership and family formation as reflecting a direct causal effect of homeownership on family formation. For instance, those who plan to start a family may also prefer homeownership. Going further, homeownership could be considered another family formation event next to cohabitation, marriage, and birth—at least in strong homeownership societies such as Britain but potentially less so in Germany, where homeownership is not normative.^11^ In robustness analyses (Online Appendix D), I partly addressed this issue by estimating models of family formation simultaneously with models of homeownership entry, and my general conclusions are consistent. Considering fertility intentions and anticipation of future births could further clarify the relationship under study (Kulu & Steele, 2013; Vidal et al., 2017).

This study offers a novel perspective by examining transitions into cohabitation. The results indicate a positive albeit small association between individual-level wealth and cohabitation among men regarding gross wealth (and debt). However, the estimated effects for women in the context of cohabitation are not statistically significant. These findings suggest the role of readily available economic resources, particularly captured by gross wealth, in enabling the establishment of an independent household with a partner, at least for men. Noteworthy, the positive association may, alternatively, be driven by wealthy partners being more attractive in the partner market. Null effects of wealth on cohabitation for women may be due to economies of scale resulting from merging two households despite the initial costs of moving together.

While there are occasional variations in the statistical significance of estimated effects between women and men, the overarching results reveal similar patterns across genders, aligning with expectations. Nonetheless, a significant and unexpected gender difference emerges, particularly for childless cohabiting women. For them, individual-level homeownership significantly increases the likelihood of transitioning to marriage and parenthood, an effect not observed when considering household-level homeownership (which also includes men’s ownership). In contrast, individual-level homeownership has no discernible impact on cohabiting men. These findings suggest that women may be more inclined to pursue transitions into marriage and parenthood when they have the security of financial independence provided by personally owning their homes. This may be particularly relevant in the German context, in which family formation for women is still often associated with labour market detachment and economic dependency. Thus, wealth in the form of homeownership may offer an alternative, independent safety net beyond the labour market and dependence on the partner.

What do these results imply about the role of wealth’s use and symbolic value and relative wealth standards for family formation? First, this study finds little evidence that relative wealth standards matter for family formation in Germany. For childless singles who transition to cohabitation and for the married who experience their first births, having above-median net wealth is associated with higher chances of transitioning, but only in separate models. The critical role of homeownership suggests that the symbolic value of wealth signalled through the owned home matters for family formation. Because homeownership also provides use value through offering accommodation, explanations drawing on the use value of wealth cannot be rejected. Nevertheless, the finding that gross wealth matters less than homeownership aligns most closely with the interpretation that the symbolic value derived from accommodation holds greater significance than its use value.

By drawing on German data, this study adds evidence from a new context to a body of research mainly focused on the USA. Overall, the economic division of family structure in Germany is less pronounced compared to the USA. Nevertheless, wealth is highly unequally distributed in Germany. At the same time, Germany is a developed welfare state with economic support for families. In this context of high inequality with strong social protection compared to other contexts with less security, e.g. the USA, where homeownership is found to be not relevant for the transition to marriage and childbirth (Schneider, 2011; Su & Addo, 2023), the symbolic value of wealth in the form of homeownership may be more important relative to its use value.

The role of homeownership in family formation must also be seen in light of recent house price inflation in Germany, which likely reduced the likelihood of young people buying property (Kholodilin & Wittenberg, 2024). In other contexts, it is argued that unaffordable housing deters fertility (Mulder & Billari, 2010), and the current results indicate that similar barriers may arise in Germany. Through public support for homeownership, such barriers may be overcome. For instance, subsidized rent-to-own programs may facilitate homeownership—particularly for less wealthy families (Gründling & Grabka, 2019).

Notwithstanding the contributions of the current study, I acknowledge some limitations. First, wealth is only measured every five years in the SOEP. By interpolating between wealth measurements, I assume wealth follows predictably linear accumulation trajectories, which may miss relevant wealth shocks and fluctuations. Second, Eastern Germany has a different demographic regime compared to Western Germany, e.g. with lower marriage rates and higher non-marital fertility (Klüsener & Goldstein, 2016). Unfortunately, the sample size is insufficient to separately examine Eastern Germany. Third, moderate multicollinearity in explanatory variables complicates the separation of their effects. Survey data is limited in clearly distinguishing aspects of wealth and must be complemented with more qualitative research on wealth and family formation. Finally, to reduce model complexity, the interdependency of family formation events could not fully be addressed in this study (Mikolai & Kulu, 2022). For instance, those with fertility intentions may be more likely to transition to marriage, which may create a spurious correlation between wealth and marriage if fertility intentions are related to homeownership preferences as argued above.

I see several avenues for future research. First, while I focus on family formation in early life, wealth can be distinctively related to union dissolution, repartnering, and higher-order fertility later in life. The role of wealth in union dissolution is more widely studied (Killewald et al., 2023; Lersch & Vidal, 2014), but Vespa (2013) is a rare study looking at the role of wealth in other later-life demographic processes. Second, I considered birth cohorts as relevant reference groups for relative wealth standards, arguing that the strong age gradient in wealth may make comparison with similarly aged others especially relevant. However, the appropriate reference group may well be another. Finally, I did not consider the complementarity or substitutability of different assets. Can particular assets substitute for the lack of other assets? Do certain assets positively interact with other assets to speed up transitions of family formation?

In conclusion, this study enhances our understanding of the relationship between wealth and family formation in Germany. The findings underscore the central importance of homeownership in facilitating the transition to marriage and childbirth, while other aspects of wealth are less central. The results suggest that the symbolic value of wealth in homeownership and its use value in providing secure accommodation play a vital role in shaping family formation.

## Data Availability

The data used in this study are available from https://www.diw.de/en/diw_01.c.678568.en/research_data_center_soep.html.

## Footnotes

1 However, wealth is positively related to intended first births and negatively associated with unintended first births, which are more likely in the USA than in Europe (Su & Addo, 2023). Also note that first childbirths in the USA tend to occur at younger ages than in many European countries, including Germany, and wealth levels can be expected to be low at young ages.

2 Insofar as first childbirths may increasingly occur in higher-order cohabitation, they are not considered in my analysis.

3 The SOEP consists of different subsamples and booster samples that have been added over time (Goebel et al., 2019).

4 Singles may have a partner who is not coresiding in the same household.

5 In supplementary analysis, I exclude respondents from Eastern Germany. Results are consistent (Online Appendix E, Figure E.2).

6 The ownership status is only imputed once.

7 All respondents with 0 (or otherwise similar) wealth were ranked equally. The computation of the median was based on the total SOEP sample before I made any analytical sample restrictions.

8 As a robustness check, I excluded interpolated values and reran analyses for frequently observed transitions. Confidence intervals are wider due to the smaller sample size and estimated effects of homeownership for the transition into cohabitation among single women turn negative (but are not statistically significant). Other results are consistent (Online Appendix E, Figure E.1).

9 To reduce model complexity in this analysis, interactions between age and time in partnership were not considered. See Carollo et al. (2023) for a potential extension to study temporal dependencies between partnership duration and age.

10 I also estimate models including individuals’ and their partners’ wealth (see Online Appendix C, Figures C.4 and C.5). For men, results are consistent. For cohabiting and married women, I find that the estimated effect of individual homeownership is slightly reduced in size and turns insignificant when their partners’ homeownership is controlled for. Despite the reduction in size, the effect remains substantial for married women’s chances of first births. The models, including individuals’ and their partners’ wealth, suffer from additional multicollinearity of these wealth variables compared to models with only individual- or household-level wealth.

11 I thank a reviewer for making this point.
